# Timing limits of ultrafast cross-luminescence emission in CsZnCl-based crystals for TOF-CT and TOF-PET

**DOI:** 10.1186/s40658-024-00663-x

**Published:** 2024-07-09

**Authors:** Katrin Herweg, Daniel Rutstrom, Vanessa Nadig, Luis Stand, Charles L. Melcher, Mariya Zhuravleva, Volkmar Schulz, Stefan Gundacker

**Affiliations:** 1 Department of Physics of Molecular Imaging Systems, Institute for Experimental Molecular Imaging, RWTH Aachen University, Aachen, Germany; 2 Scintillation Materials Research Center, University of Tennessee Knoxville, Knoxville, TN USA; 3 Department of Materials Science and Engineering, University of Tennessee Knoxville, Knoxville, TN USA; 4 Department of Nuclear Engineering, University of Tennessee Knoxville, Knoxville, TN USA; 5 Hyperion Hybrid Imaging Systems GmbH, Aachen, Germany; 6 Physics Institute III B, RWTH Aachen University, Aachen, Germany

**Keywords:** CsZnCl, Scintillator, TOF, High-frequency readout

## Abstract

**Background:**

Good timing resolution in medical imaging applications such as TOF-CT or TOF-PET can boost image quality or patient comfort significantly by reducing the influence of background noise. However, the timing resolution of state-of-the-art detectors in CT and PET are limited by their light emission process. Core-valence cross-luminescence is an alternative, but well-known compounds (e.g. BaF_2_) pose several problems for medical imaging applications, such as their emission wavelength in the deep UV. CsZnCl-based materials show promise to solve this issue, as they provide fast decay times of 1–2 ns and an emission wavelength around 300 nm.

**Results:**

In this work, we investigated two CsZnCl-compounds: Cs_2_ZnCl_4_ and Cs_3_ZnCl_5_. We validated the previously published decay times on a time-correlated single-photon counting setup with 1.786 ± 0.016 ns for Cs_2_ZnCl_4_ and 1.034 ± 0.013 ns for Cs_3_ZnCl_5_. The setup’s high resolution enabled the discovery of an additional prompt emission component with a significant abundance of 98 ± 18 (Cs_2_ZnCl_4_) and 86 ± 14 (Cs_3_ZnCl_5_) photons/MeV energy deposit. In a PET coincidence experiment, we measured the best coincidence time resolution (CTR) of 62 ps (FWHM) for Cs_2_ZnCL_4_ coupled to FBK VUV SiPMs with silicon oil. To assess the CTR for lower energies, we filtered the energy along the Compton continuum and found a deteriorated CTR that seems to be mainly influenced by photon statistics. Furthermore, this study gave us a rough estimate of e.g. 150 ps (FWHM) CTR at 100 keV energy for Cs_2_ZnCL_4_. From measurements with high activity of 14 MBq to check for pile-up effects we assume that Cs_2_ZnCl_4_ is better suited for high-rate time-of-flight applications than lutetium-based oxides. Simulations demonstrated that the stopping power of Cs_2_ZnCl_4_ is lower than for LSO:Ce,Ca, meaning that a high amount of material would be needed for TOF-PET applications. However, the stopping power seems acceptable for applications in TOF-CT.

**Conclusions:**

The fast decay time, state-of-the-art CTR in benchtop experiments and high-rate suitability make CsZnCl materials a promising candidate for time-of-flight experiments. We consider especially TOF-CT a suitable application due to its relatively low X-ray energies (~ 100 keV) and the thusly acceptable stopping power of Cs_2_ZnCl_4_. Currently, further exploration of the prompt emission and its creation mechanism is planned, as well as investigating the light transport of Cs_2_ZnCl_4_ in longer crystals.

## Introduction

In recent years, fast timing has drawn a lot of attention to improve applications using radiation detectors such as medical imaging and high-energy physics (HEP) [1, 2]. In medical imaging, specifically in time-of-flight positron emission tomography (TOF-PET) and the emerging time-of-flight computed tomography (TOF-CT) [3], the aim of fast timing is to improve patient comfort by allowing the administration of less dose or the usage of shorter scan times while keeping a constant signal-to-noise ratio (SNR). This is possible by increasing signal-to-noise ratio and detector sensitivity. To achieve beneficial fast timing in medical imaging radiation detectors, the entire detection chain has to be optimized, starting with the scintillator [4]. The coincidence timing performance of the current gold standard, lutetium-based oxides doped with Ce and co-doped with Ca (e.g. Lu$_2$SiO$_5$:Ce,Ca or “LSO”), is primarily determined by the 30–35 ns lifetime of the excited state of Ce (see Table 1) [5]. Therefore, other processes such as Cherenkov emission [2, 4, 6] and cross-luminescence (CL) [2, 7–11] are considered the most promising for faster emission of light. CL occurs in materials where the energy difference between upper core and valence band is smaller than the bandgap between valence and conduction band. If both upper core and valence band are fully populated, an electron in the upper core band is excited into the conduction band and an electron in the valence band deexcites into the upper core band in a radiative transmission to fill the resulting hole [12]. The process is of major interest due to its very fast emission with decay times below 1 ns to 2 ns [4, 12, 13]. Barium fluoride (BaF$_2$) is a well-known CL material with a fast decay time of 0.6 ns (see Table 1) and has been investigated since the eighties [13–17], but it poses many challenges such as emission in the deep-UV, medium light yield (see Table 1), medium radiation length [18] and low photofraction [19]. It has also shown considerable self-absorption of CL [14, 16] as well as a slow emission component (self-trapped exciton emission with a decay time of 630 ns) [18]. Despite the challenges presented here, there were suggestions and attempts to build fully functional PET scanners using BaF$_2$ in the eighties and nineties [14, 20, 21]. However, the work on these scanners was not continued. One reason, as stated by Ishii et al., was the low sensitivity in comparison to PET based on bismuth germanate (BGO) [20]. We believe that the sensitivity issue will need to be reevaluated, if cross-luminescent crystals can deliver sub-50ps timing resolution, as reconstruction methods would need to change. Another reason for not furthering the research in BaF$_2$ PET scanners, which Bruyndonckx et al. specifically point out in their work, is that especially the wavelength mismatch between scintillator and photosensor is an issue if better timing resolution should be achieved [21]. New developments in SiPMs, sensitive in the VUV can partially overcome this problem, but are still inferior in the deep-UV (200 nm) detection compared to the blue emission of e.g. LSO:Ce (420 nm). Furthermore, the optical coupling between scintillator and photosensor poses problems in the deep-UV [8, 9, 11].

In the last years, there have been several investigations of other CL materials, which have been discussed as fast scintillators for TOF-PET [10]. Promising candidates were found in CsCl-based compounds, such as CsZnCl [22] with an only slightly deteriorated decay time of 1 ns to 2 ns compared to BaF$_2$, as well as light emission $\gtrsim$ 300 nm wavelength (see Table 1), which could solve the wavelength mismatch. We believe the CsZnCl-based materials have the potential to reach timing resolutions, for which the sensitivity issue compared to BGO and lutetium-based crystals will have to be reevaluated.

Lately, CT has gained a lot in contrast-to-noise ratio (CNR) through the development of e.g. clinical photon-counting CT [23, 24]. As an addition to these developments, the emerging imaging method of TOF-CT was proposed to reduce patient scatter in the reconstructed CT image using time-of-flight information instead of collimators, which can significantly increase the sensitivity. CsZnCl compounds can be considered interesting scintillators for the emerging imaging method of TOF-CT [3], which needs scintillators with less stopping power than e.g. TOF-PET due to the lower radiation energy of CT. In order to realize the increased sensitivity through time-of-flight filtering, the proposed TOF-CT setup in [3] employs an ultrashort pulsed X-ray source and photodetectors with high timing resolution to extract TOF information, where the X-ray source gives a start and the signal of the photodetectors a stop time. If an X-ray were scattered in a patient, it would have a longer time of reaching the photosensor compared to a direct X-ray, making them separable if the timing resolution of the detector is high enough. A TOF-CT scanner is in need of detectors, which can be operated at high rates [3], making it difficult to use BaF$_2$ because of its additional slow emission, which would cause pile-up effects. CsZnCl compounds have not exhibited an additional slow emission, making them promising candidates for TOF-CT detectors. In this work, we investigate the timing properties of two different CsZnCl materials to determine whether they are suitable for fast timing. Additionally, we discuss how their timing properties and photon absorption properties influence their applicability in TOF-CT and TOF-PET. With Cs$_2$ZnCl$_4$ and Cs$_3$ZnCl$_5$ samples, we have conducted time-correlated single photon counting (TCSPC) measurements and coincidence time resolution (CTR) measurements with power-efficient high-frequency (HF) electronics [25] and different SiPMs. The TCSPC measurements were used to examine the scintillation emission time profile of the samples with a setup capable of higher time precision compared to previous measurements [22], while the CTR measurements were evaluated considering both regular TOF-PET energies as well as energies in the CT range. The CTR for TOF-PET energies was compared to both a lutetium-based crystal on Broadcom NUV-MT SiPMs and BaF$_2$ on FBK VUV SiPMs. Additionally, we conducted simulations to determine gamma photon absorption probability of the materials for both CT and PET energies.

**Table 1:** Material characteristics of Cs$_2$ZnCl$_4$, Cs$_3$ZnCl$_5$, BaF$_2$ and LSO:Ce,Ca, values are taken from [5, 18, 22]

	Light yield (LY) [ph/MeV]	Decay time (*τ*_*d*_) [ns]	*τ*_*d*_/LY [ps/(ph/MeV)]	Emission wavelength [nm]
Cs$_2$ZnCl $_4$	1980^a^	1.66^a^	0.90^a^	285^a^, 379^a^
Cs$_3$ZnCl $_5$	1460^a^	0.82^a^	0.71^a^	240^a^, 289^a^, 404^a^
BaF $_2$	1430^a^, 9950^b^	0.6^a^, 630^b^	0.42^a^, 63.32^b^	195^a^, 220^a^, 310^b^
LSO:Ce,Ca	38,800	36.7	0.95	420

^a^Cross luminescence

^b^Self-trapped exciton emission

## Materials and methods

**Fig. 1:**
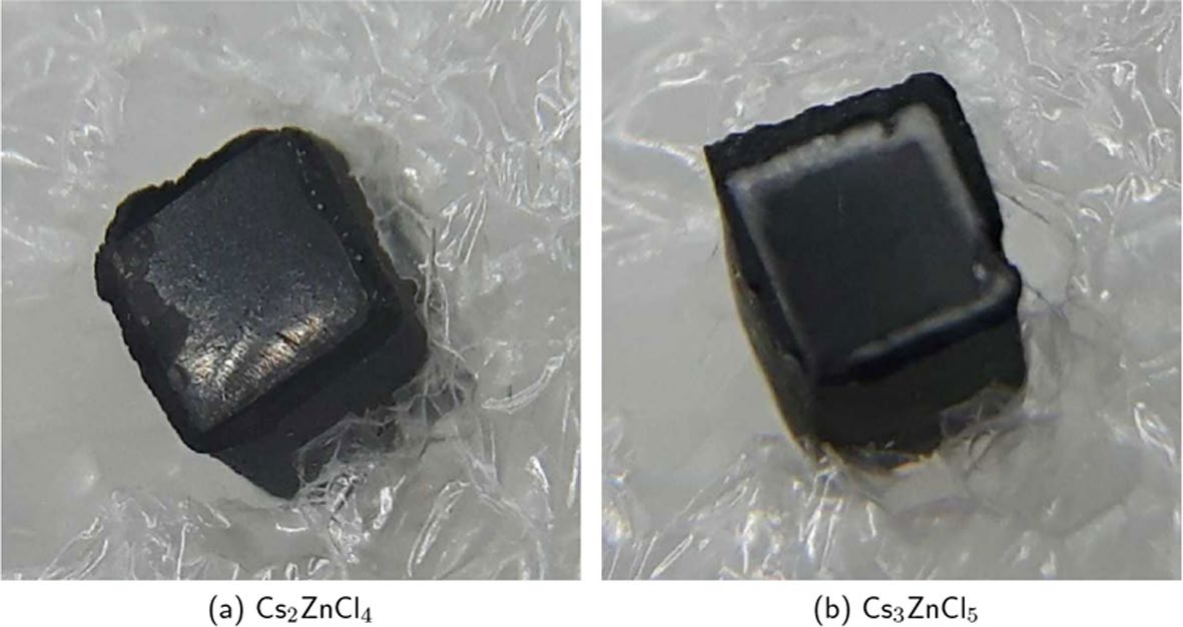
An exemplary picture of the black-painted Cs$_2$ZnCl$_4$ (**a**) and Cs$_3$ZnCl$_5$ crystal (**b**). The Cs$_3$ZnCl$_5$ crystal shows a layer of white discoloration around the edges of the crystal, which can be attributed to the binding of humidity

Single crystals of Cs$_2$ZnCl$_4$ and Cs$_3$ZnCl$_5$ were prepared at the University of Tennessee. The crystals were grown in quartz ampoules using the vertical Bridgman method. Starting raw materials were obtained from commercial vendors in the form of anhydrous beads of CsCl and ZnCl$_2$ with purities of 99.99 %, and the ternary compounds were formed via melt synthesis. Following synthesis, the ampoules were heated above the melting points of Cs$_2$ZnCl$_4$ and Cs$_3$ZnCl$_5$ and translated through a temperature gradient between 23 and 27 ${}^\circ \hbox {C}\,\hbox {cm}^{-1}$ at a rate of 0.25–0.5$\,\hbox {mm}\, \hbox {h}^{-1}$. To minimize thermally induced stress in the grown crystals, they were slowly cooled to room temperature at a rate of 3–5$\,{}^\circ \hbox {C}\,\hbox {h}^{-1}$. Additional details relating to crystal growth can be found in [22]. The grown crystals were cut into sample sizes of $2~\times ~2~\times ~3$ $\hbox {mm}^{3}$ and polished with silicon carbide polishing pads for measurement purposes. In our tests, the Cs$_2$ZnCl$_4$ sample was very stable at ambient conditions with no noticeable effects of hygroscopicity, whereas the Cs$_3$ZnCl$_5$ was slightly hygroscopic (see Fig. 1). The samples were coupled to commercial Broadcom NUV-MT SiPMs with a resin cover (active area of $3.8\times 3.8\,\hbox {mm}^{2}$, 40 $\upmu \hbox {m}$ SPADs, breakdown 32 V [26]) and VUV SiPM samples from Fondazione Bruno Kessler (FBK) [27] (active area of $3\times 3\,\hbox {mm}^{2}$, 40 $\upmu \hbox {m}$ SPADs, breakdown 32 V). While the PDE of the Broadcom SiPMs stays above 35 % down to 300 nm with a peak PDE of over 60 % at 420 nm, it declines rapidly to below 10 % for wavelengths below 300 nm [26]. For the FBK VUV SiPMs, a PDE of 22 % at 175 nm [27] and 58 % at 410 nm [9] has been reported. In combination with both SiPM types, we tested air-coupling and coupling with a silicon oil (Dow Corning 200), which has been shown to be highly transmissive down to 200 nm [8]. The crystals were measured without wrapping (naked), wrapped in several layers of Teflon or covered in black acrylic paint (water-based by Kreul). The black paint was applied by dipping the crystal into the paint.

All measurements were conducted in climate chambers with a controlled ambient temperature of 16 ${{}^\circ }\hbox {C}$. We used a $^{22}$Na source with an activity of roughly 1.7 MBq for the TCSPC and coincidence measurements.

**Fig. 2:**
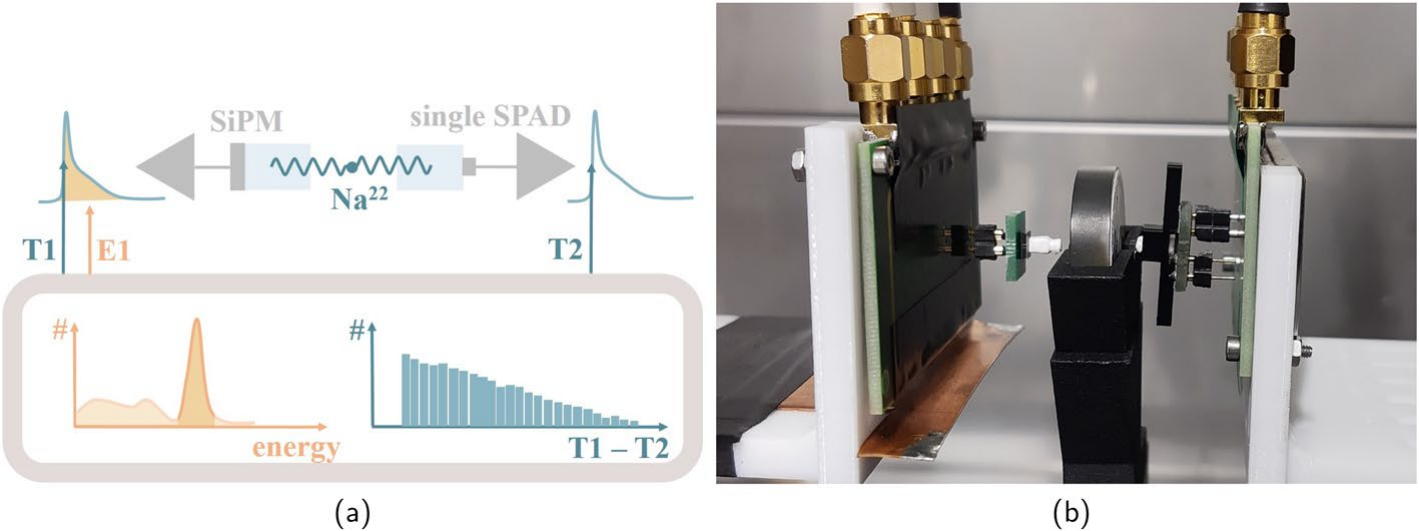
Measurement setups: **a** shows a schematic drawing of the TCSPC setup (block diagram taken from [28], licensed under CC BY 4.0), while **b** displays the setup for the CTR measurements with the reference detector on the left and the detector under test on the right

### Scintillation emission time profile

**Fig. 3:**
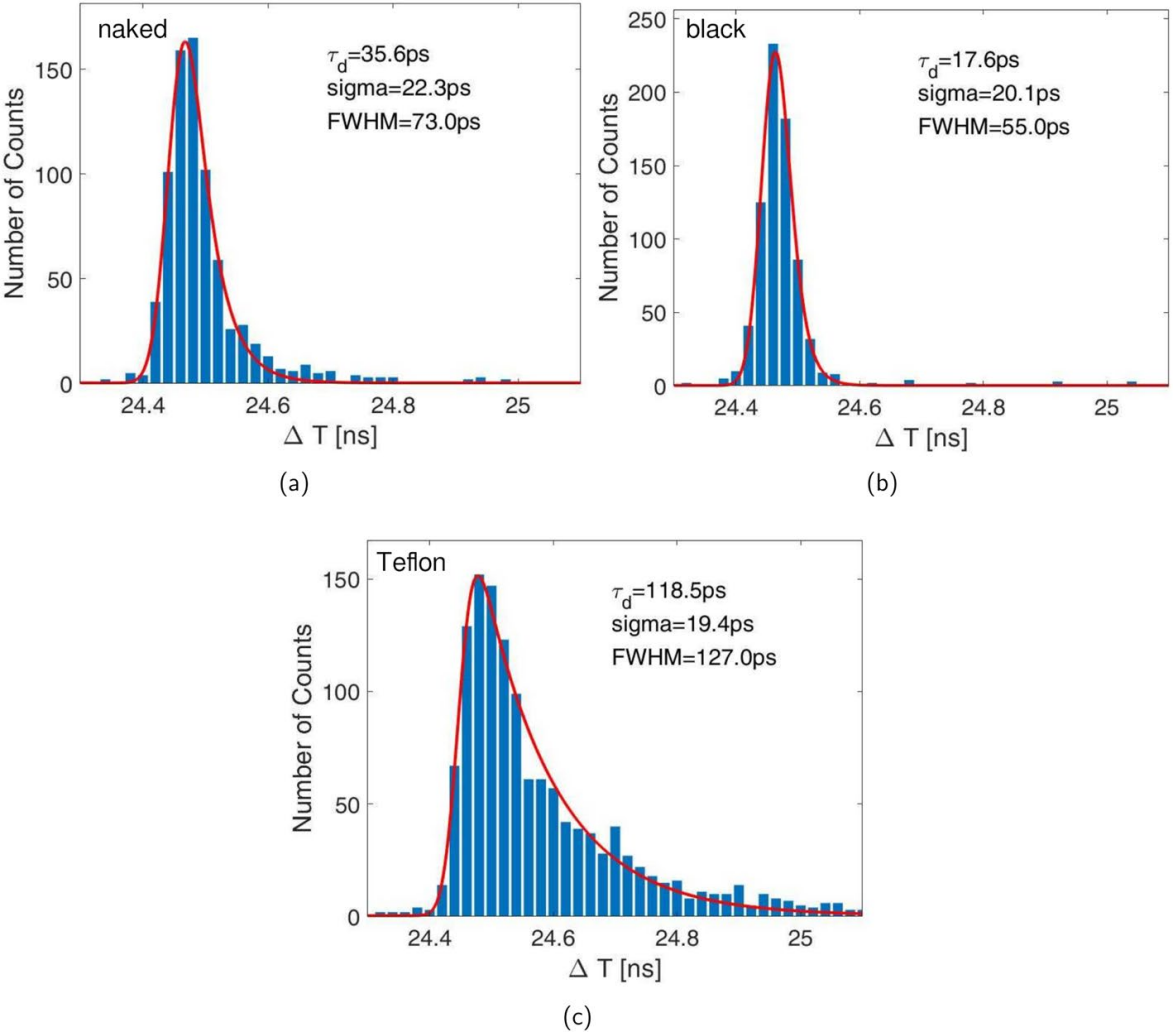
The IRF measured with a black-painted, naked and Teflon-wrapped PbF$_2$ crystal is shown in (**a**)–(**c**), respectively

The scintillation characteristics of the CsZnCl samples were measured employing a TCSPC setup, similar to [29] (see Fig. 2a). As reference a start detector with a $2~\times ~2~\times ~3\,\hbox {mm}^{3}$ lutetium-based crystal by Taiwan Applied Crystal (TAC), glue-coupled to a NUV-MT SiPM with Meltmount^™^ ($\text {n}=1.582$, Cargille), was used. This detector was set up in coincidence with a CsZnCl crystal air-coupled to a single SPAD (NUV, FBK). The impulse response function (IRF) of the system was determined by measuring the time profile of a naked, a black-painted and a Teflon-wrapped $2~\times ~2~\times ~3\,\hbox {mm}^{3}$ PbF$_2$ crystal (see Fig. 3). It can be described by a Gaussian (Eq. 1) or a Gaussian convolved with an exponential tail (Eq. 2):

1 $$\begin{aligned} IRF_{{\rm gauss}}(t) = \frac{1}{\sqrt{2 \cdot \sigma _{{\rm IRF}}}} \cdot \exp ^{- \frac{(t-\mu )^2}{2\sigma _{{\rm IRF}}}} \end{aligned}$$

2 $$\begin{aligned} IRF_{{\rm expgauss }}(t) = \frac{1}{\sqrt{2 \cdot \sigma _{{\rm IRF}}}} \cdot \exp ^{- \frac{(t-\mu )^2}{2\sigma _{{\rm IRF}}}} *\lambda \exp ^{-\lambda t} \end{aligned}$$

With $\mu$ describing the mean time delay within electronics and $\sigma _{\text {IRF}}$ being the standard deviation of the Gaussian function. The factor $\lambda$ will account for the exponential contribution of photon absorption deeper in the SPAD junction [30] or the photon transport in the crystal. In the subsequent fitting routine the IRF, with a FWHM of 55 ps (black-painted), 73 ps (naked) or 127 ps (Teflon-wrapped) was convolved with a bi-exponential function $f(t;\tau _r,\tau _{d1},\tau _{d2})$ (see Eq. 3) describing the scintillation rise ($\tau _{r}$) and decay times ($\tau _{d1}, \tau _{d2}$) [31].

3 $$\begin{aligned} f(t;\tau _r,\tau _{d1},\tau _{d2}|\theta ) = \Theta (t-\theta ) \left[ \rho _1 \frac{e^{(t-\theta )/\tau _{d1}}-e^{(t-\theta )/\tau _{r}}}{\tau _{d1}-\tau _{r}}+ \rho _2 \frac{e^{(t-\theta )/\tau _{d2}}-e^{(t-\theta )/\tau _{r}}}{\tau _{d2}-\tau _{r}}\right] \end{aligned}$$

The abundance of each decay time component is described by the weights $\rho _1$ and $\rho _2$ in equation 3. Together, both weights are constrained to add up to one, meaning we can assume the decay time abundances to be given in percent [31]. In this work the rise time $\tau _{r}$ was set to zero for all fits to a scintillation emission time profile.

### Timing performance in coincidence measurement

For the coincidence measurements, we employed an HF readout as described in [25, 32] in a coincidence setup (see Fig. 2b), using a LeCroy Waverunner 9404 M-MS (bandwidth 4 GHz, 20 GS/s) for digitization. A reference of two lutetium-based crystals ($2~\times ~2~\times ~3\,\hbox {mm}^{3}$ by TAC) glue-coupled with Meltmount^™^ ($\text {n}=1.582$, Cargille) to Broadcom NUV-MT SiPMs was established with a CTR of 58 ± 2 ps [28]. All CsZnCl measurements were conducted with a detector under test measured against the reference.

**Fig. 4:**
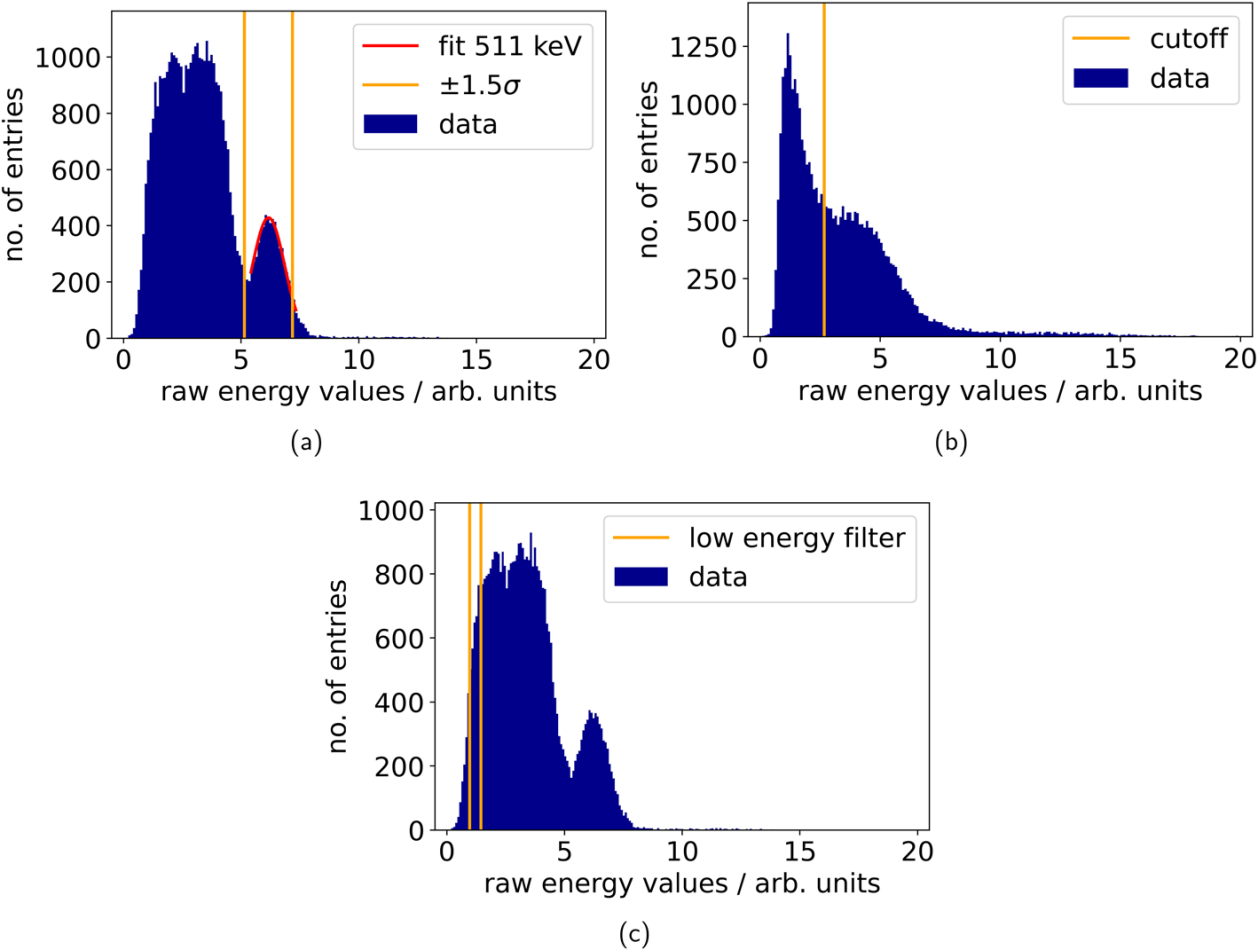
Exemplary energy spectra. The spectrum in **a** shows a well-separable photopeak with fit. In **b** the case of a not well-separated photopeak and the corresponding cut-off is demonstrated and **c** illustrates the case of a low energy filter

**Fig. 5:**
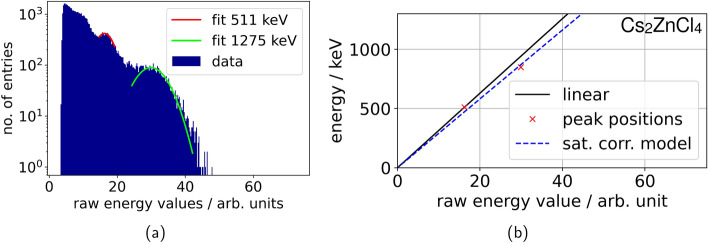
In **a** the energy spectrum of the Teflon-wrapped Cs$_2$ZnCl$_4$ crystal coupled to an NUV-MT SiPM with silicon oil is shown. The fit of the 511-keV photopeak and the 1275-keV Compton edge are marked in red and green respectively. The energy resolution of the 511-keV photopeak lies at 40 %. **b** demonstrates that the detector shows a low degree of saturation with an almost linear behaviour

For the data analysis, we conducted an energy filtering with three different methods depending on the shape of the energy spectrum and the measurement purpose. Firstly, for energy spectra with a clearly distinguishable photopeak (see Fig. 4a), we fitted a Gaussian function to the peak and selected photopeak events from the energy spectra by filtering to a $1.5\sigma$-environment around the peak position. Secondly, for energy spectra, which only showed one shoulder of the peak (see Fig. 4b), we used a cut-off, which was determined as described in [11]. We also determined the uncalibrated energy resolution for the Teflon-wrapped Cs$_2$ZnCl$_4$ crystal coupled with silicon oil to an NUV-MT SiPM to be roughly 40 %, by calculating the FWHM of the photopeak (see Fig. 5a). Additionally, we examined the saturation of the NUV-MT SiPM for the Teflon-wrapped Cs$_2$ZnCl$_4$crystal. A saturation curve was fit to the 511-keV photopeak and the 1275-keV Compton edge (848 keV) according to the model described by Schug et al [33] and showed an almost linear behaviour (see Fig. 5b).

After energy filtering the CTR is determined by fitting either one or two Gaussian functions to the time difference spectrum as described in [11]. The choice of function depended on the asymmetry of the spectrum. Finally, with the FWHM of the fitted function as CTR$_{meas}$, we correct for the reference CTR [11]. This correction step is performed for all CTRs reported in this work.

To give a first estimate for the timing performance at energies as low as 100 keV, which covers a range of higher CT energies, a dedicated energy filtering method (low energy window) is employed. For the low energy window filtering, the events of the reference detector are selected to the photopeak as described before, while for the detector under test a linear energy calibration is performed using the known position of the 511 keV peak to determine a conversion factor. According to the saturation correction, this factor is used to convert the limits of the energy window into raw energy values and only events in a variable, predetermined energy window are analyzed (see Fig. 4c). The assumption of linearity in these energy spectra is a viable approximation, as we only convert low energies. After filtering, we apply the same process as before for the time difference spectrum.

### Absorption probability simulations

To estimate the gamma photon absorption probability of CsZnCl-based crystals in TOF-CT and TOF-PET application, Geant4 simulations [34] are employed. We define Cs$_2$ZnCl$_4$ as a material and simulate gamma photons hitting the center of a crystal with a cross-section of $3 \times 3\,\hbox {mm}^{3}$ and varying length. The gamma energies range between 40 keV and 140 keV (for TOF-CT) with an additional simulation performed at 511 keV (for TOF-PET). For the determination of the gamma interaction probability the number of interacting gammas is counted and compared with the total number of gammas simulated. This calculation is also conducted solely for events, which underwent photo-effect.

## Results

### Scintillation emission time profile

**Fig. 6:**
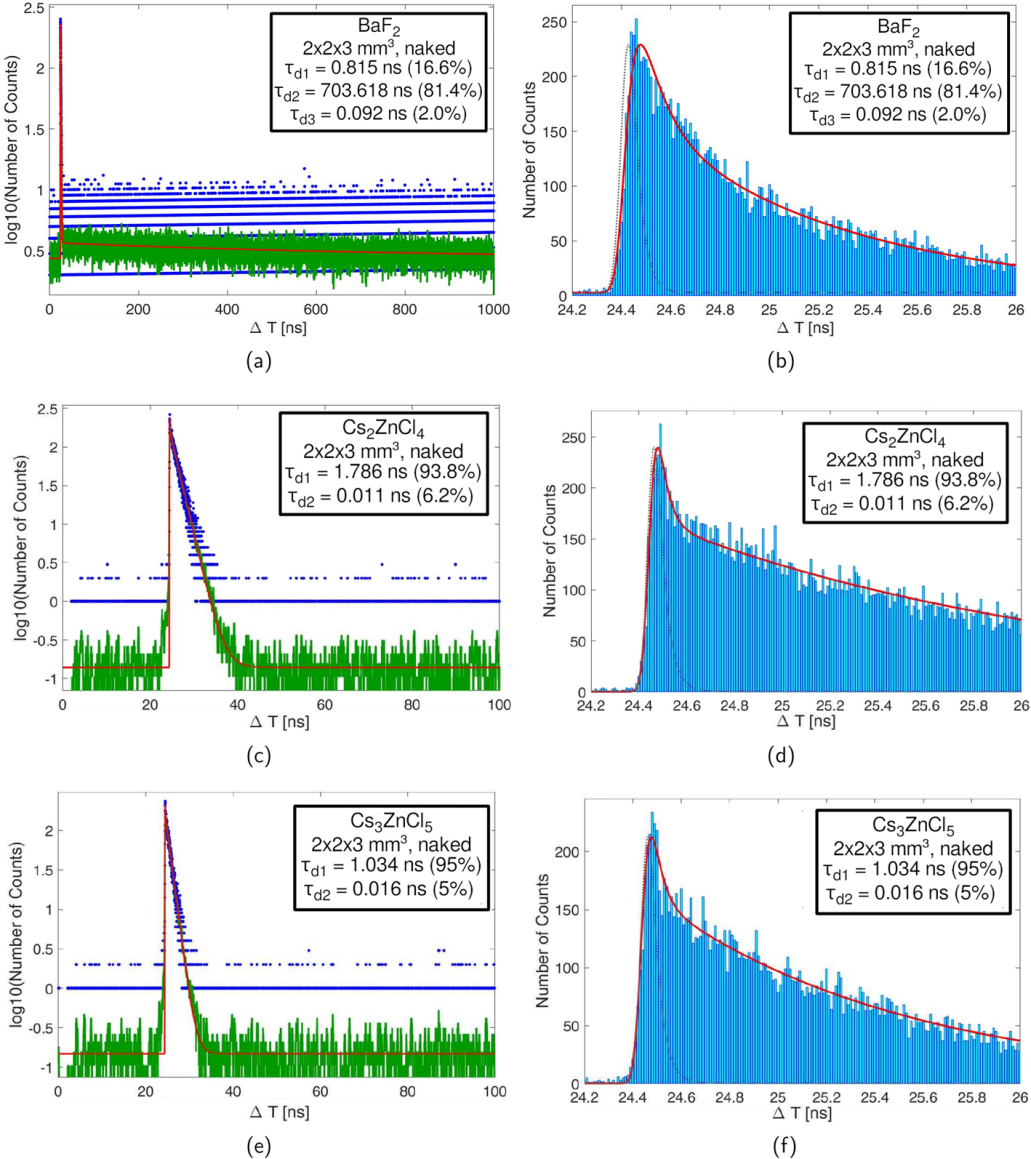
In **a** the full scintillation profile for BaF$_2$ is displayed. **b** shows a zoom into the scintillation profile of BaF$_2$. **c** and **d** show the same for Cs$_2$ZnCl$_4$ and **e** and **f** for Cs$_3$ZnCl$_5$. These measurements were conducted with naked crystals

**Fig. 7:**
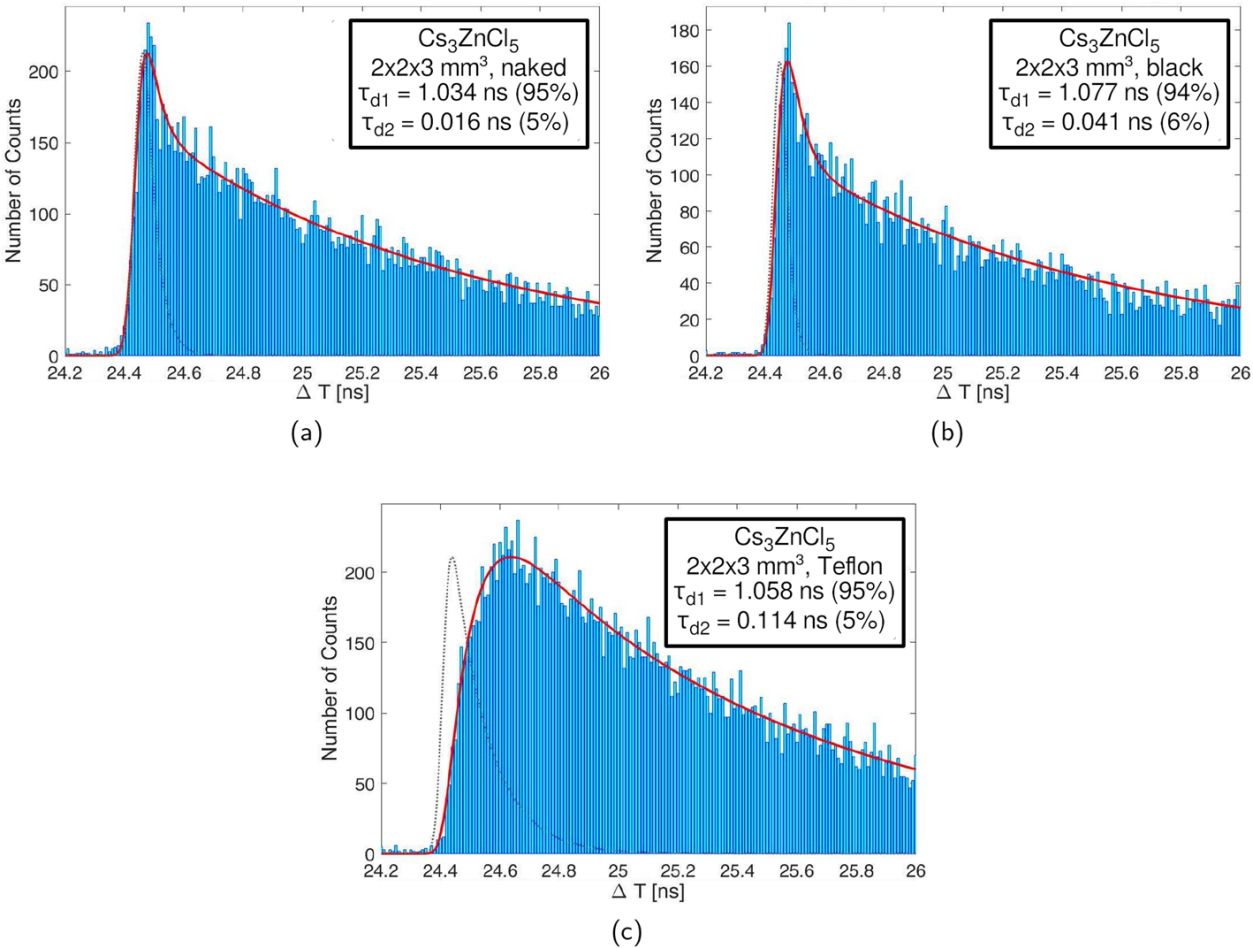
In **a** the zoomed-in scintillation profile for a naked Cs$_3$ZnCl$_5$ is displayed. **b** shows the same profile but for a black-painted crystal, while in **c** the profile of a Teflon-wrapped crystal can be seen

The scintillation emission time profile measurement of a naked BaF$_2$ crystal was performed to test our setup and showed a behavior with three decay constants with decay times of 815 ps, 704 ns and an ultra-fast decay component of 92 ps. Similar decay constants and abundances, as seen in Fig. 6a, b, were published in [9]. The CsZnCl samples display a main decay time of 1.79 ± 0.02 ns (Cs$_2$ZnCl$_4$) and 1.03 ± 0.02 ns (Cs$_3$ZnCl$_5$), respectively (see Fig. 6). Neither of the CsZnCl crystals show long tails, unlike BaF$_2$, (see Fig. 6a, c, d). However, a single decay fit was not able to reproduce the beginning of the emission time profile. Instead we applied a dual decay fit, with which we discovered a prompt component. The yield in the measurements with black-painted crystals is about 4.9 ± 0.9 % and 5.9 ± 0.9 % for Cs$_2$ZnCl$_4$ and Cs$_3$ZnCl$_5$, respectively, with a decay time of 11 ps and 16 ps (see Table 2), which can be considered prompt within the resolution of the system. Taking the light yield of both materials into account (see Table 1), this relates to $98 \pm 18$ and $86 \pm 14$ photons per MeV, respectively. Investigating the emission time profile for different wrappings of the CsZnCl samples, the decay times for both the regular decay and the prompt emission only vary slightly (see Table 2). On the other hand, a significant broadening of the emission time profile of the Teflon-wrapped Cs$_3$ZnCl$_5$ crystal can be observed (see Fig. 7c). This broadening does not affect the decay times, if the Teflon-wrapped IRF, which represents the same basic setup as the Teflon-wrapped Cs$_3$ZnCl$_5$ crystal, is used in the analysis. Cs$_2$ZnCl$_4$ shows a similar behavior.

**Table 2:** Decay times for $2 \times 2 \times 3\,\hbox {mm}^{3}$ crystals of Cs$_2$ZnCl$_4$ and Cs$_3$ZnCl$_5$ with different wrappings. Rise time in fit is set to zero

		*τ*_*d1*_ (ns)	*τ*_*d1*_ abundance (%)	*τ*_*d2*_ (ns)	*τ*_*d2*_ abundance (%)
Cs_2_ZnCl_4_	Naked	1.786 ± 0.016	93.80 ± 2.55	0.011 ± 0.013	6.20 ± 2.55
	Black	1.799 ± 0.017	95.06 ± 0.87	0.029 ± 0.011	4.94 ± 0.87
	Teflon	1.835 ± 0.022	96.54 ± 3.83	0.099 ± 0.052	3.46 ± 3.83
Cs_3_ZnCl_5_	Naked	1.034 ± 0.013	95.00 ± 2.47	0.016 ± 0.011	5.00 ± 2.47
	Black	1.077 ± 0.018	94.08 ± 0.89	0.041 ± 0.016	5.92 ± 0.89
	Teflon	1.058 ± 0.014	94.63 ± 1.69	0.114 ± 0.048	5.37 ± 1.69

### Timing performance in coincidence measurement and simulation

**Fig. 8:**
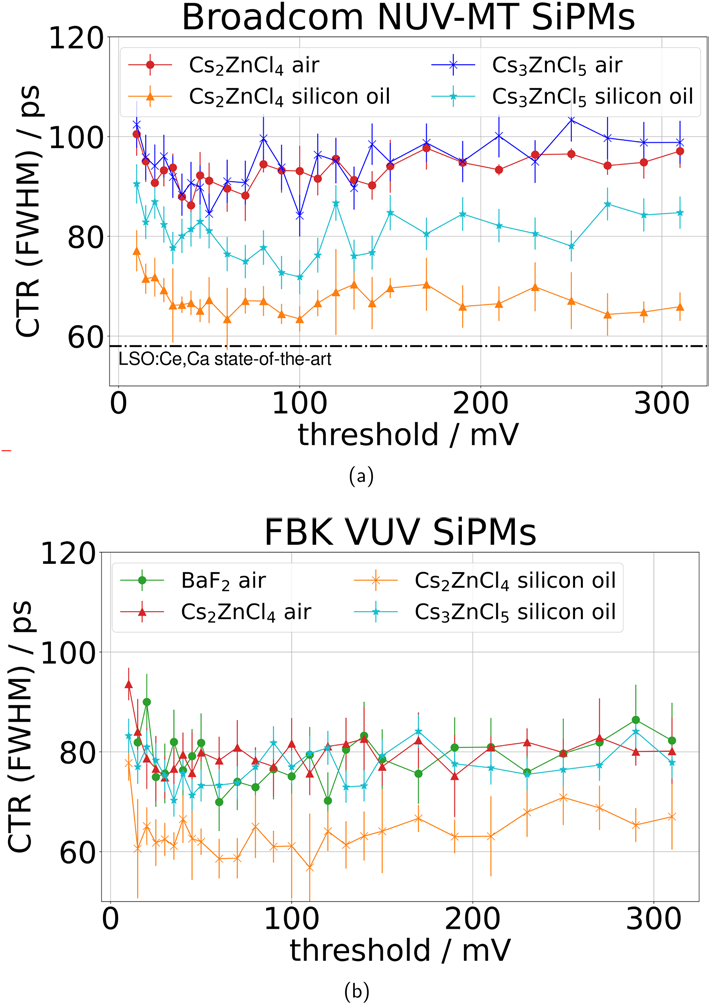
The graph displays the CTR plotted against the timing threshold. Measurements were conducted with $2 \times 2 \times 3\,\hbox {mm}^{3}$ crystals on Broadcom NUV-MT (**a**) and FBK VUV SiPMs (**b**) in a coincidence setup

The results of the CTR measurements can be seen in Fig. 8 and Table 3. Measuring both CsZnCl samples with NUV-MT SiPMs and air coupling, we achieve comparable behavior, while introducing silicon oil as a coupling medium causes a larger improvement in CTR for Cs$_2$ZnCl$_4$ than for Cs$_3$ZnCl$_5$. Using the same setup with air-coupled FBK VUV SiPMs, the timing performance of Cs$_2$ZnCl$_4$ can compare with BaF$_2$. The overall best performance is achieved at 62 ± 2 ps (FWHM) measuring Cs$_2$ZnCl$_4$ coupled to FBK VUV SiPMs with silicon oil.

Testing different wrappings or coatings for both CsZnCl materials (see Table 3), we found that Teflon wrapping did perform best for both Cs$_2$ZnCl$_4$ and Cs$_3$ZnCl$_5$. For Cs$_2$ZnCl$_4$, the timing performance decreased from Teflon over the naked crystal to the black-painted one. For Cs$_3$ZnCl$_5$ on the other hand, the performance of the black-painted one is improved compared to the naked crystal, which we attribute to the hygroscopic reaction of the crystal to the black paint as seen in Fig. 1b. The white discoloring might act similar to BaSO$_4$ or TiO$_2$ and reflect a significant amount of light back onto the SiPM. The naked crystals both show similar performance. The CTR differences between the wrappings are larger for Cs$_2$ZnCl$_4$ than for Cs$_3$ZnCl$_5$.

**Fig. 9:**
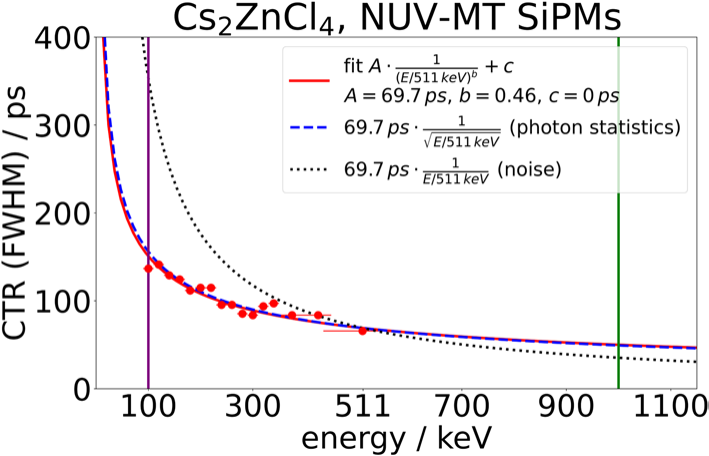
The graph displays the CTR of a $2 \times 2 \times 3\,\hbox {mm}^{3}$ Cs$_2$ZnCl$_4$ crystal wrapped in Teflon and coupled with silicon oil to a Broadcom NUV-MT SiPM. The CTR is plotted against the energy window to which the events were filtered with the low energy window filter. The energies of 100 keV and 1 MeV are marked with a purple and a green line, respectively

Choosing different energy windows of 20 keV (for the Compton continuum) and 50 keV (for the region between Compton edge and photopeak) width and also including the photopeak energy window, we made a rough estimate of the CTR along the Compton spectrum (see Fig. 9) for a Cs$_2$ZnCl$_4$ measurement with NUV-MT SiPMs and silicon oil at optimal settings (47 V bias voltage and threshold of 50 mV). The CTR was fitted with the following function with *E* being the energy detected and *c* being constrained to positive values:

4 $$\begin{aligned} f(E) = A \cdot \frac{1}{(E/511\,\textrm{keV})^{b}} + c \end{aligned}$$

For Cs$_2$ZnCl$_4$, the fit results show an amplitude *A* of 70 ps, an exponent *b* of 0.46 and an offset *c* of 0 ps. With this function, a CTR of roughly 150 ps (FWHM) can be estimated for 100 keV energy deposit. Correspondingly, we estimate a CTR of 50 ps (FWHM) for 1 MeV energy deposit. If the deterioration of the CTR for lower energies was a process purely dominated by electronic noise, we would expect it to show a 1/*E* behavior (see black dotted line in Fig. 9), while a process dominated by photon statistics would display a $1/\sqrt{E}$ behavior (see blue dashed line in Fig. 9) [4, 35]. The results for Cs$_2$ZnCl$_4$ represent the latter case, suggesting an influence of primarily photon statistics.

**Table 3:** CTR measurement results for $$2 \times 2 \times 3\,\hbox {mm}^{3}$$ crystals of Cs$$_2$$ZnCl$$_4$$ and Cs$$_3$$ZnCl$$_5$$ as well as comparison measurement with BaF$$_2 $$

	CTR (FWHM) [ps]			
	NUV-MT Air	NUV-MT Silicon oil	VUV Air	VUV Silicon oil
**Cs**$\bf _2$**ZnCl**$\bf_4$				
Teflon	91 ± 5	65 ± 3	78 ± 7	62 ± 5
Naked	−	101 ± 4	−	−
Black	−	124 ± 5	−	−
**Cs**$\bf_3$**ZnCl**$\bf_5$				
Teflon	90 ± 5	75 ± 4	−	74 ± 4
Naked	−	99 ± 4	−	−
Black$^{\dagger }$	−	86 ± 5	−	−
**BaF**$\bf_2$				
Teflon	−	−	76 ± 7	−

Energy filter is $1.5\sigma$-environment around the photopeak and CTRs are corrected for the reference CTR

$^{\dagger }$ white residue due to hygroscopicity

### Absorption probability simulations

**Fig. 10:**
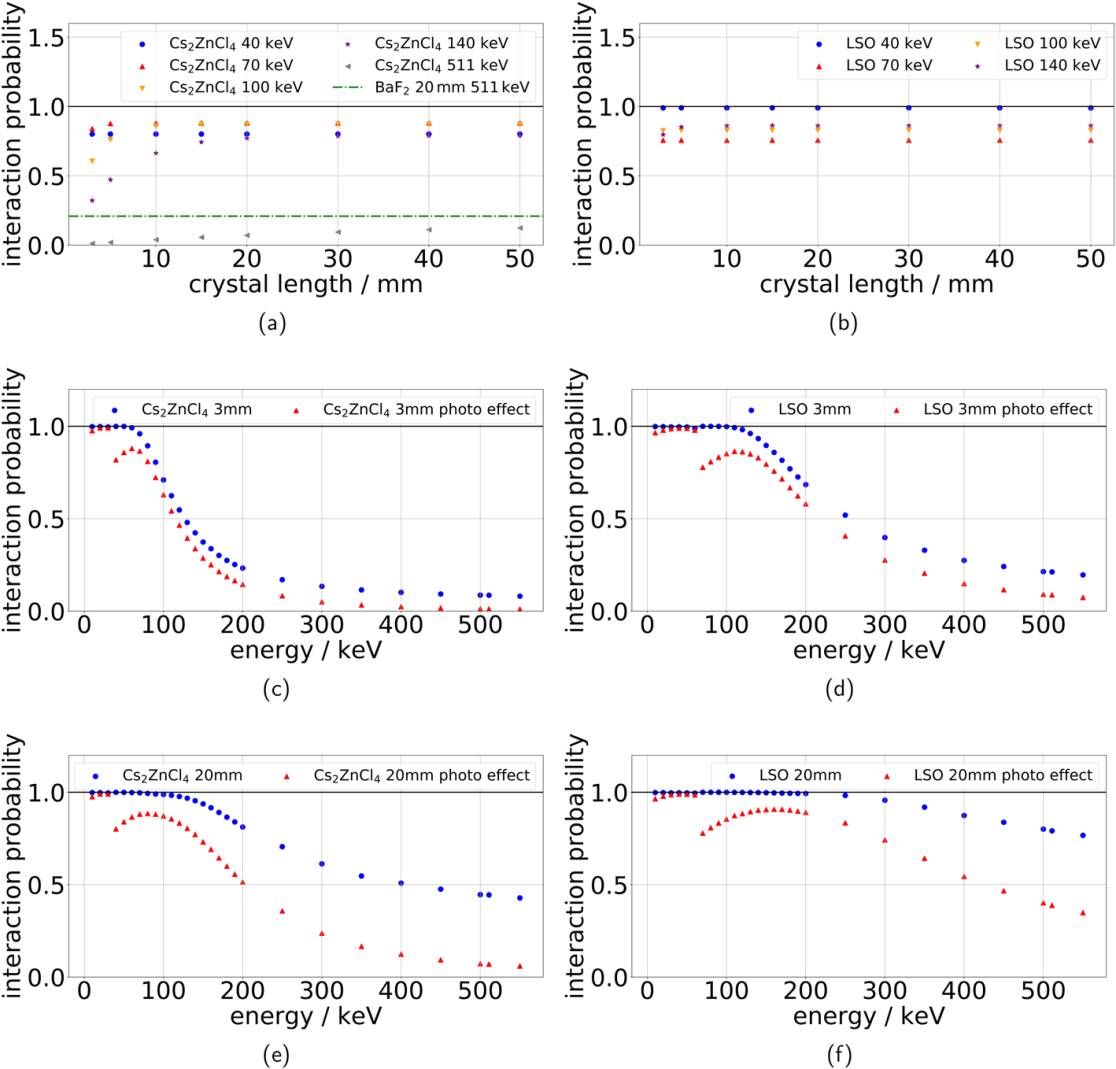
The results of the gamma photon absorption probability simulation are shown here. **a** and **b** display the absorption probability of Cs$_2$ZnCl$_4$ and LSO at the photopeak for different gamma photon energies and varied crystal lengths, while in **c** and **d** the gamma absorption probability of a 3 mm long crystal can be seen for different energies with (red) and without filter to the photopeak (blue). **e** and** f **show the gamma absorption probability of a 20 mm long crystal for different energies with (red) and without filter to the photopeak (blue)

Our simulations show, that for Cs$_2$ZnCl$_4$ crystals of lengths longer than 20 mm the low energies between 40 and 140 keV show a photo-effect interaction probability between 88 and 78 % (see Fig. 10a). For crystal lengths between 3 mm and 15 mm the results deteriorate more for the higher energies than the lower ones, ranging from 84 % (70 keV) to 32 % (140 keV) for 3 mm crystal length. While the photo-effect interaction probability at 40 keV is decreased compared to 70 keV all other energies show a trend of decreasing sensitivity with higher energy. The cause of the irregular behavior of 40 keV gammas can be seen in Fig. 10c, where a dip in the photo-effect interaction probability is visible at 40 keV. Comparing these results to LSO simulations (see Fig. 10b), the LSO interaction probability is overall larger especially for the shorter crystals. The exception at 3 mm crystal length is the simulation with 70 keV, where LSO has its own decrease in the probability of photoelectric effect (see Fig. 10d). From Fig. 10, it can be seen that for a 3 mm crystal the overall interaction probability for Cs$_2$ZnCl$_4$ declines after 60 keV, while this decline only starts at 110 keV for LSO and is less steep than for Cs$_2$ZnCl$_4$. For a longer crystal of 20 mm, the decline of the interaction probability is delayed in both crystals, though the difference in steepness behaves similarly to the 3 mm case.

The probability of photoelectric effect at 511 keV reaches a maximum of about 12 % at 50 mm crystal length for Cs$_2$ZnCl$_4$ (see Fig. 10a), which stays below the photo-effect interaction probability of a 20 mm BaF$_2$ crystal (21 %) for all crystal lengths investigated.

## Discussion

### Scintillation emission time profile

**Fig. 11:**
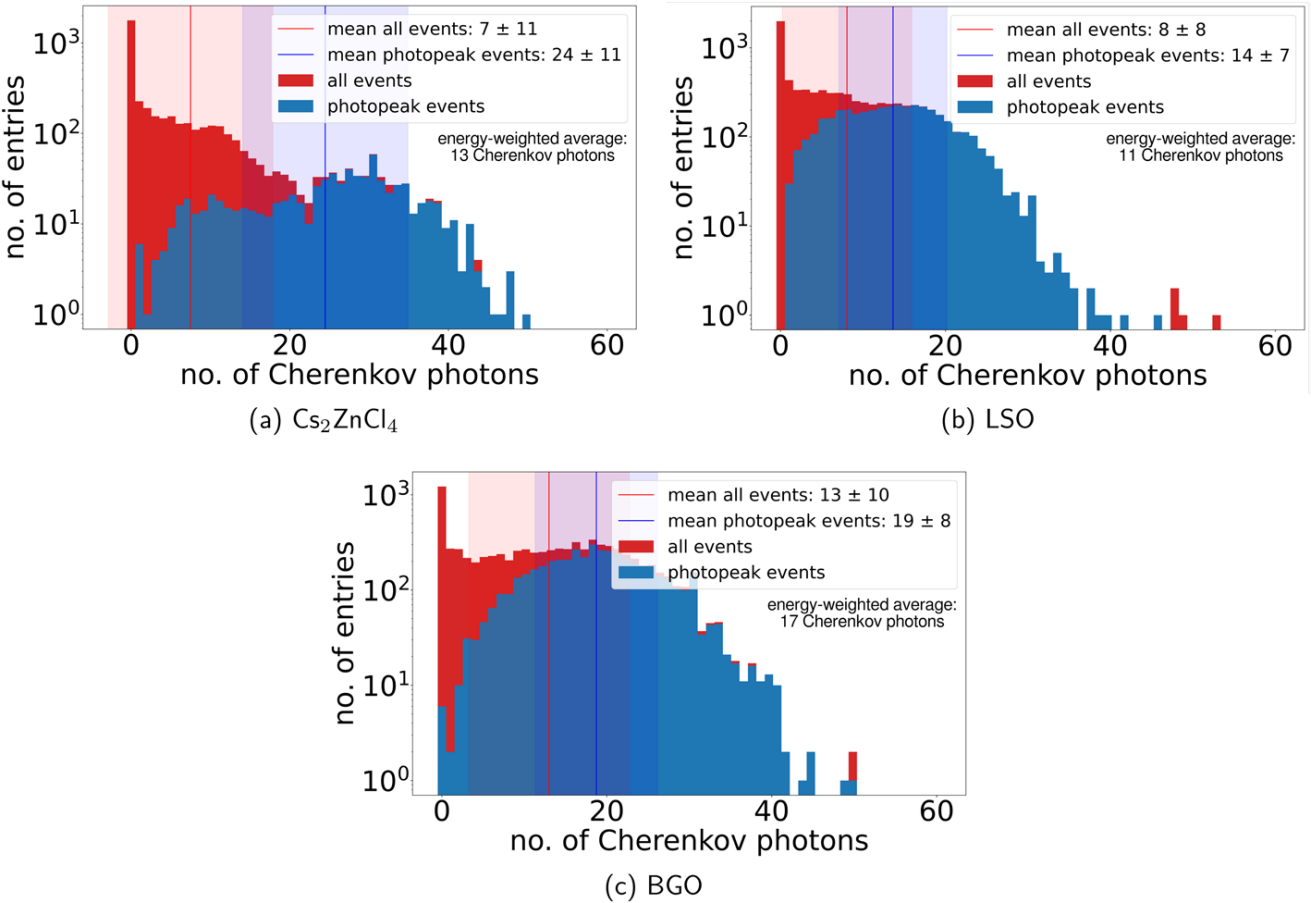
The Cherenkov yield of a Cs$_2$ZnCl$_4$, LSO and BGO crystal for impinging 511 keV gamma photons. The shaded regions display the standard deviation of the distribution

The decay times measured in this work are in accordance with the singular decay times previously published in [22] (see Table 1) as well as [7, 36]. With this short decay time of 1 ns to 2 ns and no visible long tails in the scintillation emission time profile, Cs$_2$ZnCl$_4$ and Cs$_3$ZnCl$_5$ are promising for high rate applications. The additional discovery of a prompt decay component of significant yield suggests a potential for even faster timing depending on the light transfer and collection efficiency possible within these crystals. The origin of the prompt emission is still under investigation. Via Geant4 simulations (see Fig. 11), we investigate the impact of Cherenkov radiation. The simulations show that averaging over all possible energies deposited by an impinging 511 keV gamma photon $7 \pm 11$ Cherenkov photons are produced. Considering an energy weighted average, which more closely resembles a TCSPC measurement, the mean number of produced Cherenkov photons is at roughly 13 photons per 511 keV, which is significantly lower than the yield of the prompt component for both materials (50 and 44 photons per 511 keV or 98 and 86 photons per MeV energy deposit). Therefore, we can exclude Cherenkov photons to be the sole cause of the prompt emission. At the same time, we can note, that for a photopeak event Cs$_2$ZnCl$_4$ emits on average more Cherenkov photons than both LSO and BGO, which is currently being investigated with regard to using Cherenkov photons to improve TOF-PET [6, 37, 38].

We are considering hot-intraband-luminescence in combination with Cherenkov radiation to be a potential source of this prompt emission. Another option would be quenching of the CL emission, which has been measured at lower energies for BaF$_2$ and CsCl [39, 40]. Both of this hypotheses are not tested so far and will be part of future work.

### Timing performance in coincidence measurement and simulation

The ability of Cs$_2$ZnCl$_4$ and Cs$_3$ZnCl$_5$ to perform on the same level as Ce-doped scintillators can be expected based purely on their scintillation characteristics, where the ratio of decay time and light yield shows similar values. This could be confirmed for Cs$_2$ZnCl$_4$ (see Fig. 8b) in measurements with VUV SiPMs coupled with silicon oil. In direct comparison with BaF$_2$, which shows performance similar to literature [9], Cs$_2$ZnCl$_4$ has shown that it can keep up in timing performance. We have also seen that, while the results with VUV SiPMs are overall slightly better than those achieved with NUV-MT devices, these CsZnCl-based materials can be read out successfully with commercially available NUV SiPMs, which can be handled more easily in experiment and application. The performance differences between oil-coupled Cs$_2$ZnCl$_4$ and Cs$_3$ZnCl$_5$ most likely stem from their different emission wavelengths and the corresponding light output to the photosensor.

Comparing the timing performance between naked, black-painted and Teflon-wrapped crystals, Teflon wrapping shows the best timing performance and also the highest light output. The naked crystal measurement is deteriorated by a factor of $\sqrt{2}$ compared to the Teflon measurement within a ± 1.5$\sigma$-environment. This suggests that the deterioration is mainly dependent on photon statistics. A similar deterioration was observed measuring a naked and Teflon-wrapped BaF$_2$ crystal of similar size, air-coupled to a Hamamatsu VUV SiPM. In this case, the naked crystal reached 166 ± 5 ps (FWHM) and the Teflon-wrapped crystal achieved 120 ± 4 ps (FWHM). Considering the black-painted Cs$_3$ZnCl$_5$ crystal, its performance stands out compared to the trend in the Cs$_2$ZnCl$_4$ measurements. We attribute the improved performance compared to the naked Cs$_3$ZnCl$_5$ to the slight hygroscopicity of Cs$_3$ZnCl$_5$, due to which a white film formed on the surfaces and at the edges (see Fig. 1b) of the black-painted crystal, which could better reflect light than the naked crystal.

For a first estimate of the timing performance of Cs$_2$ZnCl$_4$ at CT energies, we employed energy windows of different size scanning through the Compton continuum and fitted the time difference spectrum for each of them. The resulting CTRs show the behavior of an $1 / \sqrt{E}$ function, which would correspond to a solely photon statistical [4, 35] effect for Cs$_2$ZnCl$_4$. The photon statistical dependence for Cs$_2$ZnCl$_4$ indicates that the occurence of the prompt emission in Cs$_2$ZnCl$_4$ mentioned before is not energy dependent and seems to be a regular process of the scintillation kinematics. With an estimated CTR of 150 ps for 100 keV energy deposit, Cs$_2$ZnCl$_4$ can be expected to have good timing resolution at low energies. It would also already show a significant scatter rejection capability of between 23 % and 40 % for an object thickness of 50 mm up to between 62 % and 77 % for an object thickness of 200 mm as simulated in [3] in terms of a TOF-based CT scatter rejection.

### Absorption probability simulations

**Fig. 12:**
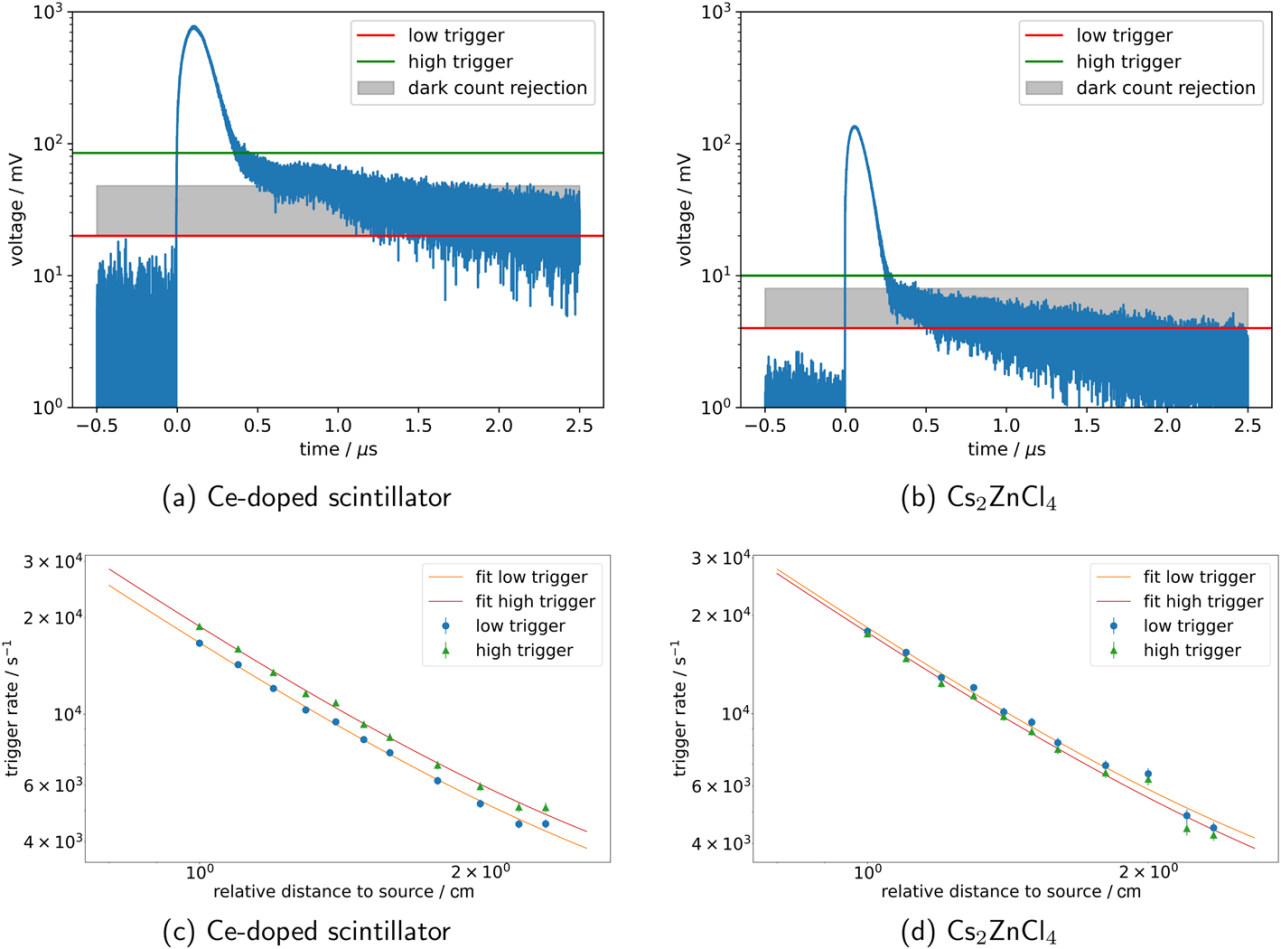
Rate measurements with 14 MBq activity for a $2 \times 2 \times 3$ ${\textrm{mm}}^{3}$ Ce-doped scintillator and a $3 \times 3 \times 3\,\hbox {mm}^{3}$ Cs$_2$ZnCl$_4$ crystal. **a** and **b** illustrate exemplary SiPM signal traces with coupled crystal, while **c** and **d** show trigger rates as a function of Na$^{22}$ source distance

Comparing the sensitivity simulations with scintillators commonly used in CT like cadmium tungstate (CdWO$_4$) and yttrium or gadolinium oxides (Y$_2$O$_3$ or Gd$_2$O$_3$), it is apparent that Cs$_2$ZnCl$_4$ scintillators cannot achieve the same stopping power of 99 % at roughly 2 mm material thickness [41] for 140 keV energy. For the same energy, an interaction probability of 95 % is reached at 20 mm crystal length for Cs$_2$ZnCl$_4$ (see Fig. 10e). 3 mm long Cs$_2$ZnCl$_4$ crystals only reach a maximum of 32 % absorption probability without filtering to photoeffect events. So larger volumes of material would be needed to reach the same stopping power as state-of-the-art CT scintillators. Considering how the timing performance would change for a larger crystal length, we did coincidence measurements as described in Sect. 2 with a 20 mm long Cs$_2$ZnCl$_4$ crystals, with which we reached a preliminary CTR of 170 ±7 ps (FWHM). While the need for large Cs$_2$ZnCl$_4$ crystals might seem disadvantageous, the crystals reach a decay time of 1 ns to 2 ns, which is a factor of $10^{3}$ or more smaller than what regular CT scintillators usually achieve [41]. This should prevent pile-up effects, which need to be avoided in order to prevent image artefacts in regular CT scanners [41]. In a test, measuring with 14 MBq $^{22}$Na sources, we could support this assumption by showing that Cs$_2$ZnCl$_4$ crystals display relatively few pile-up events compared to the tested Ce-doped scintillator. We set two trigger thresholds to a measured waveform (see Fig. 12a, b and calculated how many signals surpassed each of the thresholds, then measured this trigger rate for different distances from the radioactive source. The trigger rates over the relative distance to the source change with a $\frac{1}{r^2}$ dependency for both Cs$_2$ZnCl$_4$ and the Ce-doped scintillator. After fitting this dependency with a variable amplitude and offset, we could show that both amplitude and offset of the Cs$_2$ZnCl$_4$ trigger are the same within a $1\sigma$-environment (see Fig. 12 and Table 4).

Considering the simulation results for 511 keV, it can be assumed that a substantial amount of material would be needed to achieve a sensitivity comparable to state-of-the-art LSO detectors in TOF-PET application, which might speak against using Cs$_2$ZnCl$_4$ in this context, depending on the self-absorption and DOI capability of the crystal. Another aspect influencing the applicability in TOF-PET, especially in regards to large quantities of material, is the producibility and production cost of the crystals. However, currently, it is difficult to assess the production cost given that these materials are still in the early research stage. If we compare them to similar, commercially available halide scintillators, there are no anticipated roadblocks regarding raw materials costs. Likewise, their low melting points (600 ${{}^\circ }\hbox {C}$) are advantageous from a manufacturing standpoint and allow the use of conventional low-cost growth methods. The main challenges will be associated with the crystal yield (i.e. ability to grow large-volume crack-free crystals). This is an ongoing development, but so far, preliminary scale up efforts have been promising for crystal sizes upwards of 22 mm in diameter.

**Table 4:** Fit results of high-rate measurements of Cs$_2$ZnCl$_4$ and a Ce-doped scintillator. Low and high trigger values were fit with $A\cdot \frac{1}{r^2}+b$

	Amplitude A (mm^2^ s^−1^)		Offset b (s^−1^)	
	Low trigger	High trigger	Low trigger	High trigger
Cs$_2$ZnCl $_4$	16,650 ± 509	16250 ± 477	1700 ± 223	1465 ± 209
Ce-doped scintillator	15130 ± 225	17010 ± 268	1577 ± 101	1790 ± 124

## Conclusion and outlook

In this work, we validated and measured more precisely previously published decay times of Cs$_2$ZnCl$_4$ and Cs$_3$ZnCl$_5$ [22]. During these measurements, we found a prompt emission with significant abundance yielding about 100 prompt photons per MeV energy deposit. We excluded Cherenkov radiation as the sole cause for this emission and plan to further investigate its origin. Additionally, Geant4 simulations and wrapping studies will be conducted, as the light transport in the crystal will become one of the main limiting parameters for optimal timing.

The timing performance of Cs$_2$ZnCl$_4$ is on par with state-of-the-art Ce-doped crystals and best values measured with BaF$_2$. At the same time, it has the advantage of keeping a comparable performance coupled to both commercially available SiPMs with protective resin from Broadcom and VUV SiPMs from FBK. The best CTR is achieved for Cs$_2$ZnCl$_4$ coupled to an FBK VUV SiPM with silicon oil at 62 ± 2 ps. This is close to the state-of-the-art TOF-PET performance with Ce-doped crystals at a CTR of 58 ps [4]. For a first estimate of timing performance at CT energies a low energy filter was applied to the Cs$_2$ZnCl$_4$ energies, scanning over the Compton continuum. This allowed to predict a possible CTR of 150 ps for an energy deposit of 100 keV. In simulation studies, Cs$_2$ZnCl$_4$ showed low stopping power for 511 keV gammas relevant for PET, but suitable stopping power for energies in the CT range. Further measurements in the form of DOI scans with 20 mm Cs$_2$ZnCl$_4$ crystals are planned to investigate the light transport and to give answers for an effective minimization of the scintillation light transport time spread.

## Data Availability

The datasets used and/or analysed during the current study are available from the corresponding author on reasonable request.

## Abbreviations

TOF-CT: Time-of-flight computed tomography
TOF-PET: Time-of-flight positron emission tomography
HEP: High-energy physics
LSO: Lutetium oxyorthosilicate
CL: Cross-luminescence
BaF_2_: Barium fluoride
BGO: Bismuth germanate
TCSPC: Time-correlated single photon counting
CTR: Coincidence time resolution
HF: High-frequency
LY: Light yield
*τ*_*d*_: Decay time
FBK: Fondazione Bruno Kessler
TAC: Taiwan Applied Crystal
IRF: Impulse response function
*τ*_*r*_: Rise time
CdWO_4_: Cadmium tungstate
Y_2_O_3_: Yttrium oxide
Gd_2_O_3_: Gadolinium oxide
